# Parliament and the Professions

**Published:** 1920-04-24

**Authors:** 


### Parliament and the Professions.

### Indian Degrees in British East Africa.

MR. WATERSON asked the Under-Secretary of State for the Colonies if he is aware that persons holding Indian University medical and law degrees, such as L.M. and S. and B.A.B.L., who are eligible to practise medicine and law respectively in India, are not allowed to so practise in British East Africa. Colonel Amery stated that Indian medical degrees or diplomas are recognised in East Africa Protectorate, provided that they entitle the holder to registration in the United Kingdom.

### Optical Appliances.

MR. SITCH asked the Lord-President of the Council if it is his intention to appoint a Committee to inquire into the supply of spectacles and eyesight glasses generally by vendors not qualified by examination, on the same lines as those of the recent inquiry into unqualified dental practitioners.

Dr. Addison, who replied, said that the recent inquiry was not limited in scope to the unqualified practice of dentistry, and the conditions prevailing, which rendered it necessary, have no parallel in the circumstances connected with the production of optical appliances.

